# Ubiquitination-based Classification and a Prognostic Signature Identify the Role of TRIM21 in Sarcoma Progression

**DOI:** 10.2174/0109298673388379251113061745

**Published:** 2026-01-15

**Authors:** Lin Zhang, Weihao Lin, Jinhui Liu, Yuheng Hong, Zheng Cao, Zhentao Yu, Xiaoli Feng, Yibo Gao

**Affiliations:** 1 Department of Thoracic Surgery, National Cancer Center/National Clinical Research Center for Cancer/Cancer Hospital & Shenzhen Hospital, Chinese Academy of Medical Sciences and Peking Union Medical College, Shenzhen, 518116, China;; 2 Department of Thoracic Surgery, Nanfang Hospital, Southern Medical University, Guangzhou, 510515, China;; 3 Department of Thoracic Surgery, National Cancer Center/National Clinical Research Center for Cancer/Cancer Hospital, Chinese Academy of Medical Sciences and Peking Union Medical College, Beijing, 100021, China;; 4 Department of Pathology, National Cancer Center/National Clinical Research Center for Cancer/Cancer Hospital, Chinese Academy of Medical Sciences and Peking Union Medical College, Beijing, 100021, China;; 5 Central Laboratory & Shenzhen Key Laboratory of Epigenetics and Precision Medicine for Cancers, National Cancer Center/National Clinical Research Center for Cancer/Cancer Hospital & Shenzhen Hospital, Chinese Academy of Medical Sciences and Peking Union Medical College, Shenzhen, 518116, China;; 6 Laboratory of Thoracic Oncology & Department of Thoracic Surgery, National Cancer Center/National Clinical Research Center for Cancer/Cancer Hospital, Chinese Academy of Medical Sciences and Peking Union Medical College, Beijing, 100021, China;; 7 State Key Laboratory of Molecular Oncology, National Cancer Center, National Clinical Research Center for Cancer, Cancer Hospital, Chinese Academy of Medical Sciences and Peking Union Medical College, Beijing, 100021, China;; 8 Laboratory of Translational Medicine, National Cancer Center/National Clinical Research Center for Cancer/Cancer Hospital, Chinese Academy of Medical Sciences and Peking Union Medical College, Beijing, 100021, China

**Keywords:** Sarcomas, ubiquitination, TCGA, tumor microenvironment, prognosis, TRIM21

## Abstract

**Introduction:**

Sarcomas are heterogeneous mesenchymal tumors with poor responses to systemic therapies. Ubiquitination is a post-translational modification that regulates various physiological processes and cancer growth.

**Methods:**

We analyzed the transcriptomic data of 256 sarcoma patients from The Cancer Genome Atlas (TCGA) to define the network and prognostic value of predefined ubiquitination-related genes and performed subgroup analyses. Additionally, the role of TRIM21 in sarcoma progression was explored using cellular experiments.

**Results:**

We identified two ubiquitination-related clusters, and patients in the two clusters were characterized by different survival outcomes, enriched pathways, and characteristics of the tumor microenvironment. A ubiquitination-related signature involving LRRC41, RNF125, TRIM21, and UBE3D was identified to predict prognoses. The signature was associated with patient prognoses in the public sarcoma cohorts and our independent cohort. Immunohistochemistry analyses revealed that the risk score and CD8 could distinguish patients with different survival outcomes in our cohort. Mechanistically, different risk groups were also characterized by distinct enriched pathways and characteristics of the tumor microenvironment. Cellular experiments revealed that TRIM21 overexpression could suppress tumor progression in sarcomas.

**Discussion:**

This study provided a novel classification for sarcoma patients based on expressions of ubiquitination-related genes. The classification and the prognostic model may facilitate the understanding of sarcoma pathogenesis, the prediction of prognosis and immunotherapy response for sarcoma patients. Meanwhile, it was confirmed that TRIM21 suppressed sarcoma progression and identified it as a potential target for therapeutic interventions.

**Conclusion:**

The classification and signature stratify sarcoma patients for prognosis and immunotherapy response, with TRIM21 representing a promising therapeutic target.

## 1 INTRODUCTION

Sarcomas represent a heterogeneous group of rare malignant tumors that originate from mesenchymal tissues and can be divided into soft-tissue and bone sarcomas [1]. Although accounting for only a small proportion (<1%) of all malignant tumors, they are responsible for a significant loss of life in that they are among the most aggressive and common tumors in adolescents [2, 3]. To date, the primary management strategy for localized disease generally involves surgery and is often complemented by perioperative radiotherapy and/or chemotherapy; despite receiving optimal local therapy, approximately 40% of sarcoma patients eventually develop metastatic disease, which frequently leads to lethal outcomes [2]. Unfortunately, systemic treatment options are very limited and have a low response rate in unresectable disease, and significant toxicities often limit treatment dosing and length of therapy [2, 3]. Furthermore, sarcomas consist of more than 100 subtypes, with each subtype reacting differently to systemic therapies [2]. Therefore, identification of sarcoma patients with different prognoses and treatment sensitivities will improve the understanding of these diverse tumors and facilitate individualized therapies.

Ubiquitination and its related cellular processes are fundamental cellular mechanisms that regulate diverse aspects of cell biology, such as protein degradation, activation, subcellular localization, and molecular interactions [4]. Ubiquitination could be achieved by conjugating to the lysine residues of substrate proteins by an enzymatic cascade, including E1 activation enzymes, E2 conjugation enzymes, and E3 ubiquitin ligases [5]. The ubiquitination process could also be reversed by deubiquitinating enzymes that remove ubiquitin from substrates [6]. Studies have shown that dysregulation of the ubiquitination system plays a key role in many diseases, including neurodegenerative diseases, autoimmune diseases, and cancers [6].

Interactions between the ubiquitin system and cancers have been reported in a lot of studies recently. For example, ubiquitination acts as a central regulator of LAG3’s immunosuppressive function in cancer, and activates LAG3 to suppress T cell responses for suppressing anti-tumor immunity [7]; TRIM21 is an E3 ligase and promotes tumor growth and gemcitabine resistance in pancreatic cancer by inhibiting EPHX1-mediated arachidonic acid metabolism [8]; PRMT5-mediated arginine methylation stabilizes GPX4, a key regulator of ferroptosis, by blocking the binding of the Cullin1-FBW7 E3 ligase complex to inhibit GPX4 ubiquitination and proteasomal degradation, therefore inducing ferroptosis resistance in cancer cells [9]. However, the roles of Ubiquitin-Related Genes (URGs) in sarcomas have not been fully elucidated yet. A deeper understanding of the underlying mechanisms may facilitate future research on sarcomas. In this study, we integrated transcriptional and clinical data of The Cancer Genome Atlas (TCGA) sarcoma cohort (TCGA-SARC) to systematically explore the prognostic values of URGs in TCGA-SARC patients. We identified two distinct ubiquitin-related clusters that were characterized by distinct patient prognoses, enriched pathways, and tumor environment; further, we identified a ubiquitin-related prognostic signature that could robustly predict the prognoses and immunotherapy of sarcoma patients. In addition, the predictive value of the signature was validated in a sarcoma cohort from our center, and the suppressive role of TRIM21 in sarcomas was explored. Hopefully, the characterization of different clusters and establishment of the model could help to better understand sarcoma pathogenesis and facilitate prognostic management and risk stratification for sarcoma patients.

## 2 MATERIALS AND METHODS

### 2.1 Data Source

The gene expression matrices of sarcomas with paired clinical data were downloaded from The Cancer Genome Atlas database (https://portal.gdc.cancer.gov/). After excluding normal samples, relapsed or metastatic tumors, 256 cases were included in the final analyses. The clinical data and gene expression files of 85 osteosarcoma patients derived from the osteosarcoma cohort from the TARGET database (TARGET-OS, (https://ocg.cancer.gov/programs/target/projects/osteosarcoma) were used as a validation cohort. GSE17674 microarray data on 44 Ewing sarcoma cases were downloaded from the GEO database (https://www.ncbi.nlm.nih.gov/geo/), and these patients were used as another validation cohort. The data were normalized and log2(X+1) transformed.

### 2.2 Data Extraction

The list of 807 URGs was obtained from the iUUCD 2.0 database (http://iuucd.biocuckoo.org/) [10]. After matching with the mRNA expression profiles from the TCGA-SARC dataset, 802 URGs were obtained. The genes were then filtered with the standard of >1 transcripts per kilobase, and the remaining 752 genes were incorporated into the final analyses (Table **S1**). The enrichment analyses of these genes were performed and shown in Figs. (**S1A** and **S1B**).

### 2.3 Screening for Sarcoma Subtypes and Enrichment Analysis

Based on the expression profiles of URGs, molecular subtypes were identified using the Nonnegative Matrix Factorization (NMF) R package, as described in previous research [11]. The package was run with the following settings: “brunet” option and 100 iterations. The optimal cluster number was determined according to the cophenetic, dispersion, and silhouette coefficients. Gene Set Variation Analyses (GSVA) were performed with the GSVA R package to identify the pathways correlated with the different sarcoma subtypes. The gene set of “h.all.v7.2.symbols” was downloaded from MSigDB and used for enrichment analysis [12].

### 2.4 Tumor Microenvironment Analysis

The Epithelial-Mesenchymal Transition (EMT) score was calculated based on the EMT gene signature, as previously described [13]. Key immune characteristics [14], including leukocyte fraction, stromal fraction, SNV neoantigen, Indel neoantigen, T-Cell Receptor (TCR) Shannon, and TCR richness, were obtained from the website (https://gdc.cancer.gov/about-data/publications/panimmune) and were compared between distinct ubiquitin-related clusters or between high and low risk groups. Immune infiltration analyses were performed using TIMER 2.0 (http://timer.comp-genomics.org/), with the CIBERSORT algorithm, absolute mode [15, 16].

### 2.5 Identification and Evaluation of the Ubiquitin-related Prognostic Signature

Univariate Cox analysis and random survival forest analysis were performed subsequently to identify the ubiquitin-related prognostic signature, as previously described [13]. The risk score was calculated using the formula: risk score = ∑(Ci×Expi); ‘C’ stands for the coefficient of genes and ‘Exp’ is their expression levels. All the patients in different cohorts were divided into high and low-risk groups based on their median risk scores. Time-dependent Receiver Operator Characteristic (ROC) curves were drawn to evaluate the prognostic prediction efficacy of the model.

### 2.6 Sample Collection

This study was conducted with the approval of the Independent Ethics Committee of Cancer Hospital, Chinese Academy of Medical Sciences (NCC2021C-052). We retrospectively enrolled patients with pathologically confirmed sarcoma, available FFPE tissue for IHC, and complete baseline clinical and follow-up data. Those with missing data, lost to follow-up, or inadequate tissue were excluded. Finally, specimens from 50 treated sarcoma patients were obtained from Cancer Hospital, Chinese Academy of Medical Sciences. Given the retrospective nature of the research, the committee granted a waiver of informed consent. Patients’ information was summarized in Table **S2**.

### 2.7 Immunohistochemistry and Quantification

Immunohistochemistry and quantification were performed following previously established methods [17]. The primary antibodies used were rabbit anti-LRRC41 (dilution 1:20; Proteintech, 20457-1-AP), rabbit anti-RNF125 (dilution 1:20; Proteintech, 13290-1-AP), rabbit anti-TRIM21 (dilution 1:200; Proteintech, 12108-1-AP), rabbit anti-UBE3D (dilution 1:100; Abcam, ab121927) and mouse anti-CD8 (dilution 1:4000; Proteintech, 66868-1-Ig). The secondary antibodies were peroxidase-labeled anti-rabbit IgG (H+L) antibody (dilution 1:200; SeraCare, 5220-0336) and peroxidase-labeled anti-mouse IgG (H+L) antibody (dilution 1:200; SeraCare, 5220-0341). Protein expression levels were quantified using the H-score method, as described in previous studies [17, 18]. The H-score was calculated by multiplying two parameters: the percentage of positive cells (0: 0-10%, 1: 11%-40%, 2: 41-70%, 3: 71%-100%) and the staining intensity (0: very weak, 1: weak, 2: moderate, 3: strong). Two pathologists (Z.C. and X.F.), blinded to the patients’ clinical data, independently validated the results.

### 2.8 Cell Culture and Plasmid Transfection

Human sarcoma cell lines, U2OS, and A673, were procured from Procell Life Science&Technology Co., Ltd., Wuhan, China [19]. U2OS cells were incubated in McCoy's 5A (Procell, Wuhan, China), and A673 cells were incubated in DMEM (Gibco, CA, USA), both were supplemented with antibiotics and 10% of fetal bovine serum (Corning, NY, USA) at 5% CO_2_ and 37°C. Negative control vectors or plasmids encoding TRIM21 were purchased from Tsingke Biotechnology Co., Ltd (Beijing, China). The vectors and plasmids were transfected into the tumor cells using Lipofectamine 3000 (Invitrogen, CA, USA) as per the manufacturer’s instructions.

### 2.9 CCK8 and Colony Formation Assays

The CCK8 and colony formation assays were conducted following established protocols, which were discussed in a similar study [20]. In the CCK8 assay, tumor cells were plated in a 96-well plate, and cell proliferation was assessed using the CCK8 reagent (CK04; Dojindo, Kumamoto, Japan). Absorbance at 450 nm was measured with a microplate reader at specified time intervals, as per the manufacturer’s guidelines. For the colony formation assay, cells were seeded in 60 mm plates and incubated for 10-12 days. After fixation with 4% formaldehyde in phosphate-buffered saline, the colonies were stained with 2% crystal violet solution, followed by imaging.

### 2.10 Statistical Analysis

Data analysis and figure plotting were conducted using R software v. 3.6.1, GraphPad Prism 8.0, and Stata 16.0. For group comparisons, the Wilcoxon test and the Kruskal-Wallis test were employed in comparison between two and three groups, respectively. Univariate Cox regression analyses were executed using the corresponding R packages. Differences in survival outcomes across clusters or risk groups were assessed through the Kaplan-Meier method and Log-rank tests, implemented with the R packages “survminer” and “survival”. Statistical significance was defined as a two-tailed *p*-value < 0.05.

## 3 RESULTS

### 3.1 Identification and Characterization of Two Ubiquitin-related Subgroups

**Fig. (1):**
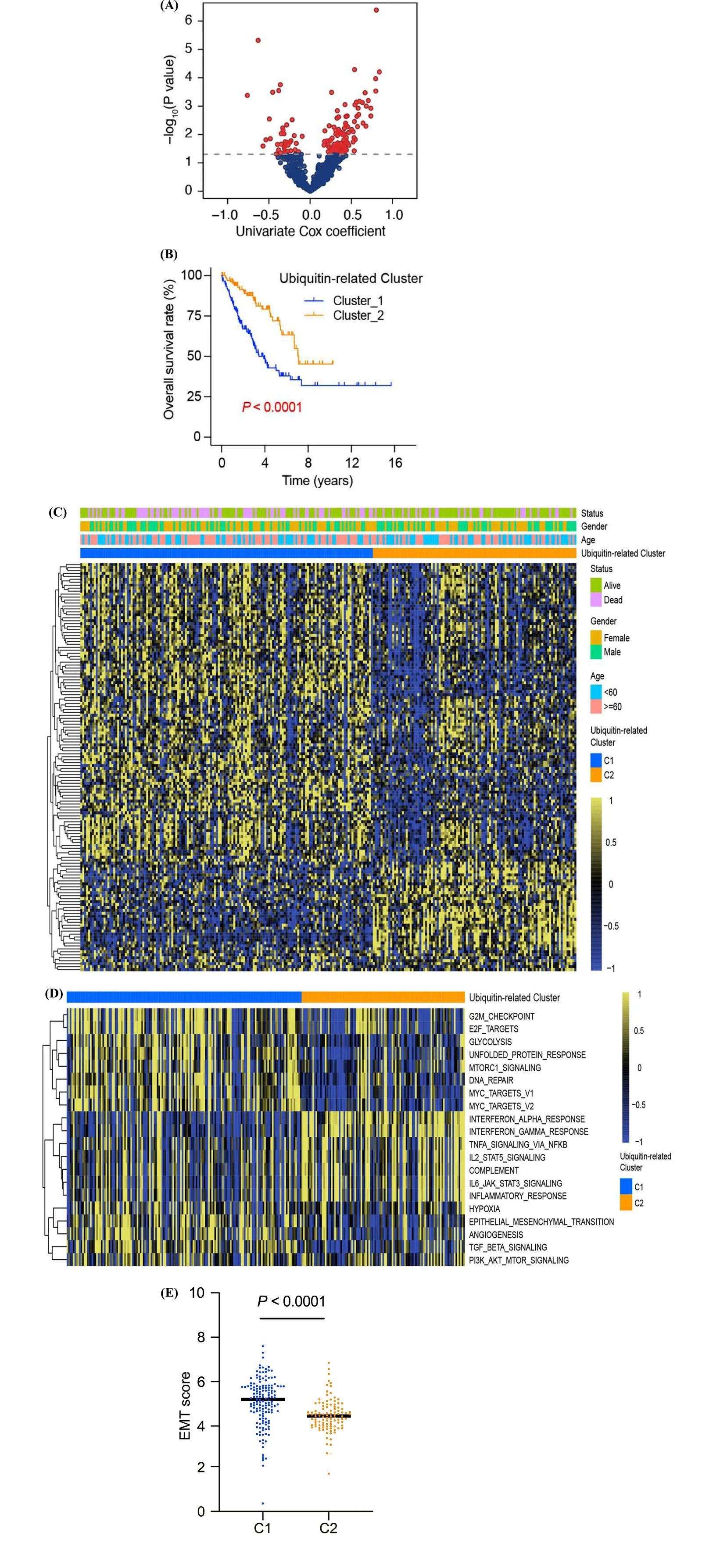
Identification of two URG-based clusters with distinct prognoses and biological processes in sarcomas. (**A**) Volcano plot showing univariate analyses of the URGs in TCGA-SARC. (**B**) Kaplan-Meier overall survival analyses of patients in different clusters. (**C**) Heatmap showing the expressions of URGs in patients with different survival status, gender, and age. (**D**) Gene set variation analysis of Hallmark pathways in different clusters. (**E**) Comparison of EMT scores in different clusters. URG, ubiquitin-related gene; TCGA-SARC, The Cancer Genome Atlas sarcoma cohort; EMT, epithelial-mesenchymal transition.

The detailed workflow was shown in Fig. (**S2**). Among 752 URGs, 138 prognostic-related URGs were identified by univariate Cox analyses (Fig. **1A**, Table **S3**). NMF clustering was performed to classify sarcoma patients based on the expression of 138 URGs using TCGA-SARC. K = 2 was selected as the optimal cutoff based on the NMF algorithm (Fig. **S3A** and **S3B**), which means that patients from TCGA-SARC were classified into two clusters. A survival analysis revealed that the Overall Survival (OS) of patients in cluster 2 was better than that in cluster 1 (*p* < 0.0001; Fig. **1B**).

We further examined the relationship between the two clusters and clinicopathological characteristics in the TCGA cohort. The heat map illustrates the association between clinical features and the expression levels of URGs in clusters 1 and 2 (Fig. **1C**). The enriched pathways were also analyzed by GSVA (Table **S4**), and the results were shown in a heat map in Fig. (**1D**). We observed that the EMT pathway was enriched in cluster 1 (Fig. **1D**). Consequently, we estimated the EMT scores for both clusters and found that cluster 1 exhibited a significantly higher EMT score (Fig. **1E**). The findings suggested an activated immune response for cluster 2, which might account for its better survival, and was explored in subsequent analyses.

### 3.2 Characterization of the Tumor Microenvironment of the Two Clusters

**Fig. (2):**
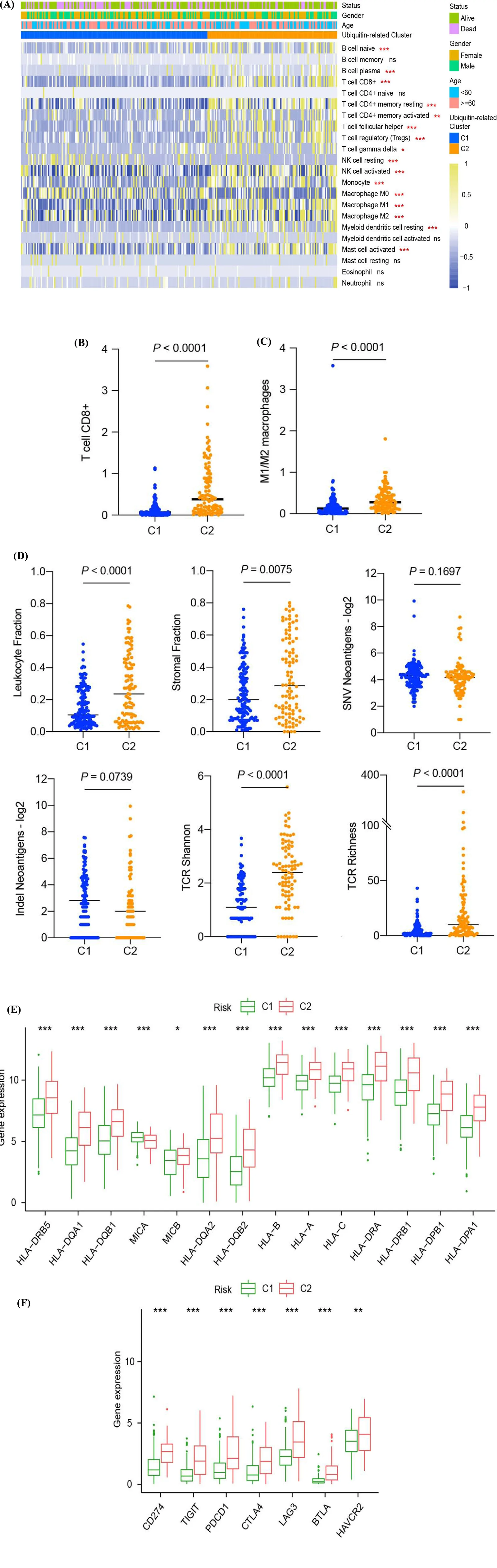
Comparison of the tumor microenvironment between two ubiquitin-related genes-based clusters. (**A**) Heatmap showing the abundance of different immune cell types in the two clusters, as estimated by CIBERSORT. (**B, C**) Comparison of CD8^+^ T cell infiltration levels and M1/M2 macrophage proportions between the two clusters. (**D**) Comparison of leukocyte fraction, stromal fraction, SNV neoantigens, indel neoantigens, TCR Shannon, and TCR richness between the two clusters. (**E, F**) Comparison of antigen presentation-related gene expressions and prominent checkpoints gene expressions between the two clusters. *p < 0.05, **p < 0.01, ***p < 0.001.

To understand the characteristics of the immune infiltration, we performed CIBERSORT. The distribution of immune cells (listed in Table **S5**) and their relationship with some clinical parameters of the two clusters were shown in a heat map in Fig. (**2A**). Specifically, cluster 2 was characterized by higher infiltration levels of CD8^+^ T cells and a higher M1/M2 macrophage proportion (Figs. **2B**, **C**).

We then evaluated the expressions of key immune characteristics (including leukocyte fraction, stromal fraction, single-nucleotide variant neoantigens, indel neoantigens, TCR Shannon, and TCR richness) and Major Histocompatibility Complex (MHC) class II genes. Leukocyte fraction, stromal fraction, TCR Shannon, and TCR richness were significantly higher in cluster 2 (Fig. **2D**). Meanwhile, most of the MHC class II genes were also higher in cluster 2 (Fig. **2E**).

The expression of several prominent checkpoints, including CD274, TIGIT, PDCD1, CTLA4, LAG3, BTLA, and HAVCR2, was also evaluated. The expressions of these checkpoints were all significantly higher in cluster 2 (Fig. **2F**), indicating the possibility of a better response to immunotherapy for this cluster.

### 3.3 Identification and Evaluation of a Ubiquitin-related Prognostic Signature

From 752 URGs, 59 candidate genes with significant prognostic values (*p* < 0.01) were identified by univariate analyses (Fig. **S4A**). Random survival forest analyses were subsequently conducted to refine the pool of candidate genes, and the top 10 genes, ranked based on their importance, were selected for combination analysis and model construction (Fig. **S4B**). The top 10 combinations with the smallest *p* values were shown in Fig. **S4C**. Among them, the combination with the least gene number (LRRC41, RNF125, TRIM21, and UBE3D) was selected for building the final model. The risk score was calculated based on the following formula: risk score = 0.58705789*LRRC41 + (-0.2270716)*RNF125 + (-0.5404087)*TRIM21 + 0.41875036*UBE3D. The prognostic values of the four genes were investigated, and all of them showed a significant prognostic value in patients from TCGA-SARC (Fig. **S4D**).

**Fig. (3):**
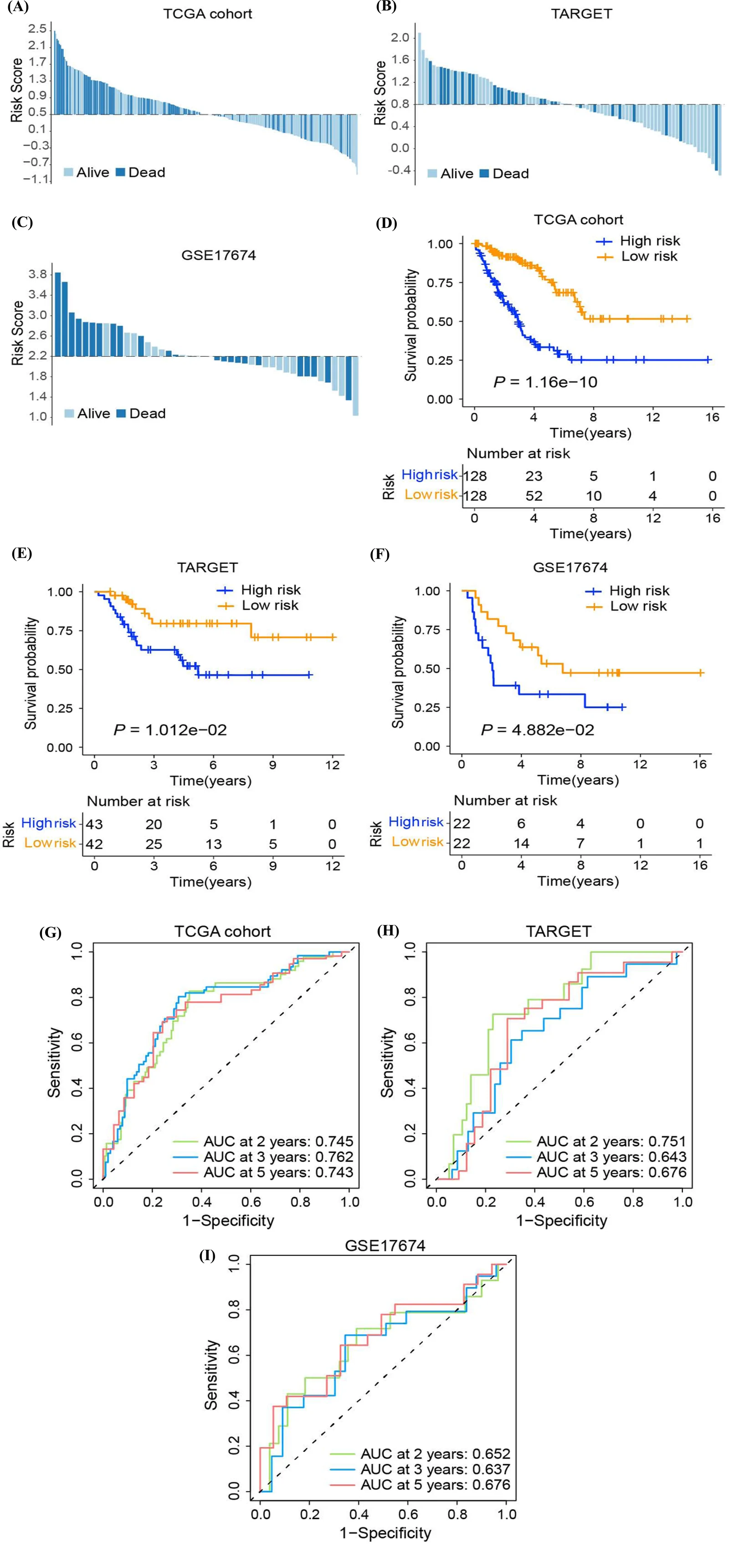
Identification and validation of the ubiquitin-related prognostic model in public datasets. (**A-C**) Distribution of the risk scores of patients in the datasets. (**D-F**) Kaplan-Meier survival analyses of patients from different risk groups in the datasets. (**G-I**) Receiver operating characteristic curves evaluating the predictive accuracy of the model in the datasets.

Further, the prognostic values of the model were explored in three public datasets. First, the patients in TCGA-SARC (training cohort) were assigned to a high- risk or a low-risk group according to the median risk score (Figs. **3A**-**I**). The survival analysis revealed that patients in the low-risk group had significantly better OS (Fig. **3D**). The areas under the ROC curve for OS were 0.745 at 2 years, 0.762 at 3 years, and 0.743 at 5 years (Fig. **3G**). The risk scores were then calculated likewise for patients from two validation cohorts (Fig. **3B** for TARGET-OS and Fig. **3C** for GSE17674), and patients in low-risk groups all had significantly better prognoses (Figs. **3E**, **F**). Analyses of the 2-year, 3-year, and 5-year prognostic prediction abilities indicated that the model had relatively strong robustness and effectiveness (Figs. **3H**, **I**).

### 3.4 Pathway Analyses and Tumor Microenvironment Characterization in Different Risk Groups

**Fig. (4):**
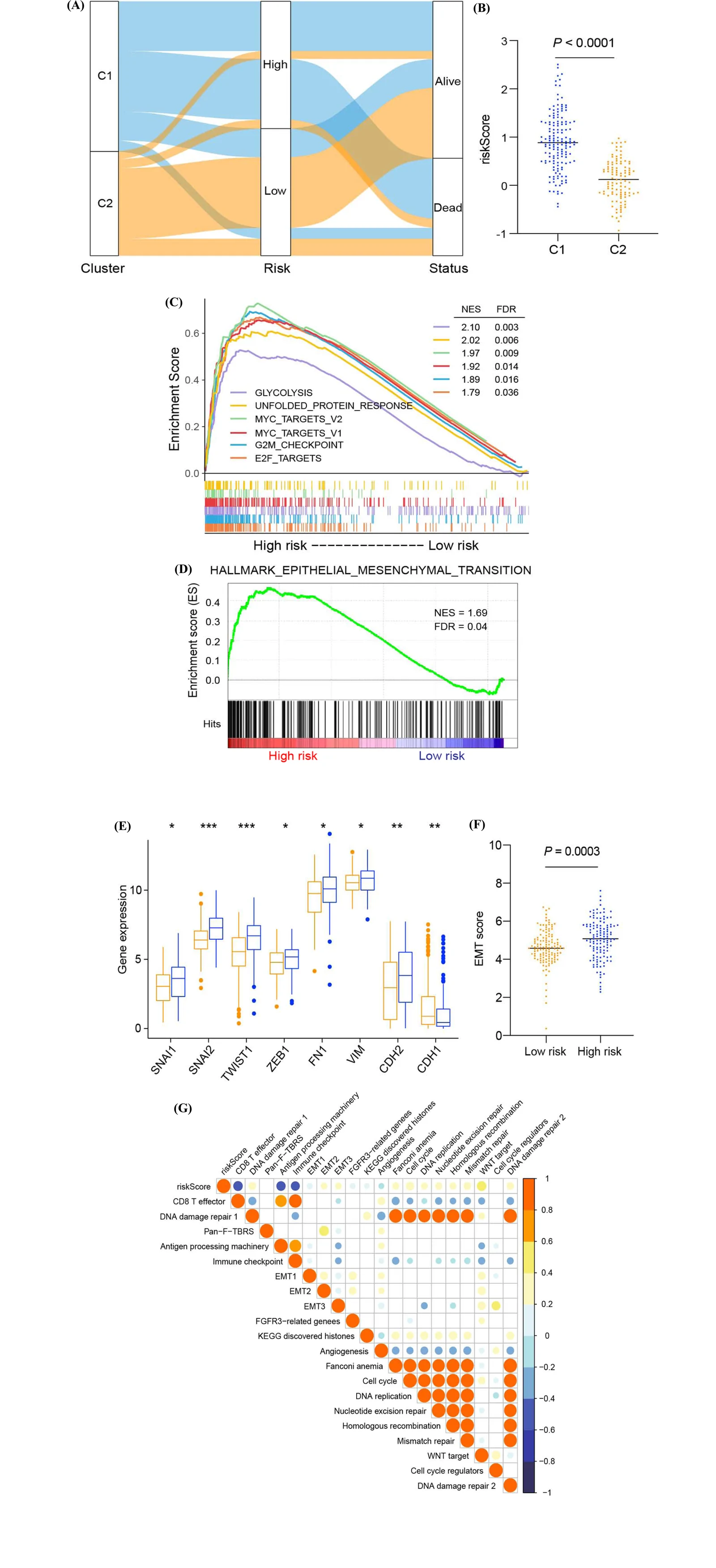
Characterization of patients from different risk groups in TCGA-SARC. (**A**) Alluvial diagram depicting relations of ubiquitin clusters, different risk groups, and survival status. (**B**) Comparison of risk scores between the two clusters. (**C**) GSEA of Hallmark pathways in different risk groups. (**D**) GSEA of the Hallmark EMT in different risk groups. (**E, F**) Comparisons of the expressions of EMT-related genes and EMT scores between the two risk groups. (**G**) Correlations between the model and the published gene signatures in TCGA-SARC patients. GSEA, gene set enrichment analyses; TCGA-SARC, The Cancer Genome Atlas sarcoma cohort; EMT, epithelial-mesenchymal transition. *p < 0.05, **p < 0.01, ***p < 0.001.

We explored the correlations between the clustering system and the prognostic model. Changes in the attributions of individual sarcoma patients from TCGA-SARC in different clusters, risk subgroups, and survival status were shown in an alluvial diagram; the diagram showed that cluster 1 patients had the highest proportion of the high-risk subtype and were linked to a higher incidence of fatality (Fig. **4A**). Correspondingly, the mean risk score of patients in cluster 1 was significantly higher than that of cluster 2 (Fig. **4B**). We then further investigated the enriched pathways related to the model. GSEA revealed that pathways including glycolysis, the unfolded protein response, MYC targets V2, MYC targets V1, the G2M checkpoint, and E2F targets were enriched in the high-risk group (Fig. **4C**). Interestingly, we noticed that Hallmark EMT was also enriched in the high-risk group (Fig. **4D**). Therefore, differential expressions of EMT-related genes were explored in the two groups. Among 8 EMT-related genes, 7 were significantly higher in the high-risk group, while 1 (CDH1) was lower in that group (Fig. **4E**). Consistently, the EMT score was significantly higher in the high-risk group (Fig. **4F**). GSVA (Gene Set Variation Analysis) was also performed, revealing a correlation between the risk score and known biological processes. (Table **S6**). Interestingly, the risk score was negatively correlated with the CD8 T effector pathway, antigen processing machinery, and immune checkpoint (Fig. **4G**). Based on this, relative pathways were further explored.

**Fig. (5):**
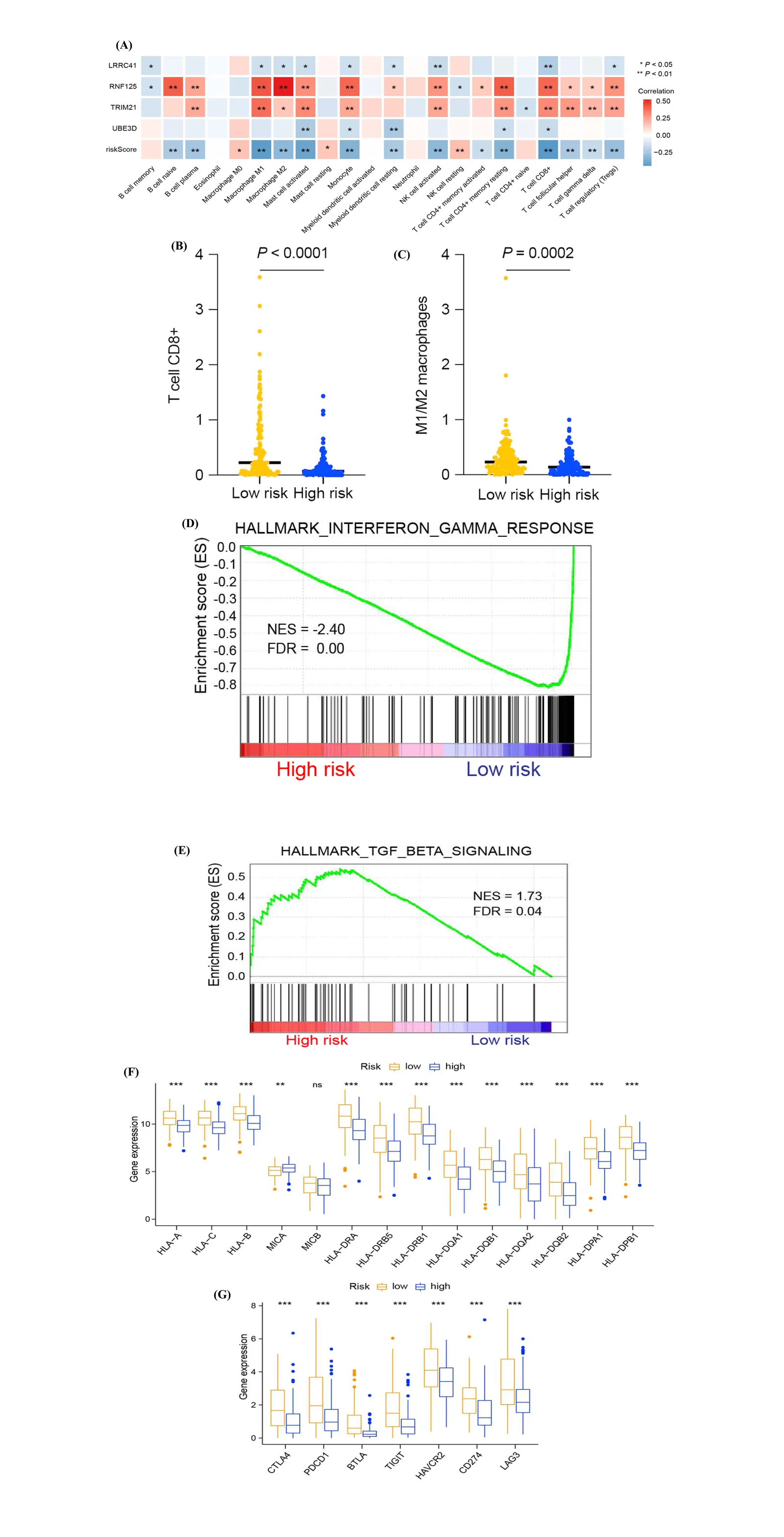
Characterization of the tumor environment between different risk groups. (**A**) Correlation heatmap of immune cell infiltration levels with the expression of the four signature genes and the risk score. (**B, C**) Comparisons of CD8^+^ T cell infiltration levels and M1/M2 macrophage proportions between different risk groups. (**D, E**) Enrichment plots of gene set enrichment analyses of the Hallmark interferon-gamma response and TGF-beta signaling in different risk groups. (**F, G**) Comparison of antigen presentation-related gene expressions and prominent checkpoints gene expressions between different risk groups. *p < 0.05, **p < 0.01, ***p < 0.001; ns, not significant.

To further understand the underlying mechanisms, the tumor microenvironment of the two risk groups was characterized. Firstly, Spearman’s correlation analyses were performed to explore the correlations between the risk score, each gene in the model, and immune cell infiltration. A widespread correlation was observed between the expression of the genes, the risk score, and immune cell infiltration (Fig. **5A**). Specifically, CD8^+^ T cells and M1/M2 macrophages were significantly higher in the low-risk group (Figs. **5B** and **C**), which is consistent with the GSVA result. Consistent with this, GSEA revealed an enrichment of the Hallmark interferon gamma response in the low-risk group and an enrichment of the Hallmark TGF beta signaling in the high-risk group (Figs. **5D**, **E**). According to the GSVA result, we further tested the major MHC class II genes, and most of them showed higher expression in the low-risk group (Fig. **5F**). Additionally, the expression of several prominent checkpoint genes in different risk groups was examined, and significantly higher levels of CTLA4, PDCD1, BTLA, TIGIT, HAVCR2, CD274, and LAG3 were observed in the low-risk group (Fig. **5G**). The results suggest that patients in the low-risk group are characterized by an inflamed microenvironment and may have a better response to immunotherapy.

### 3.5 Validating the Prognostic Signature in Our Cohort

**Fig. (6):**
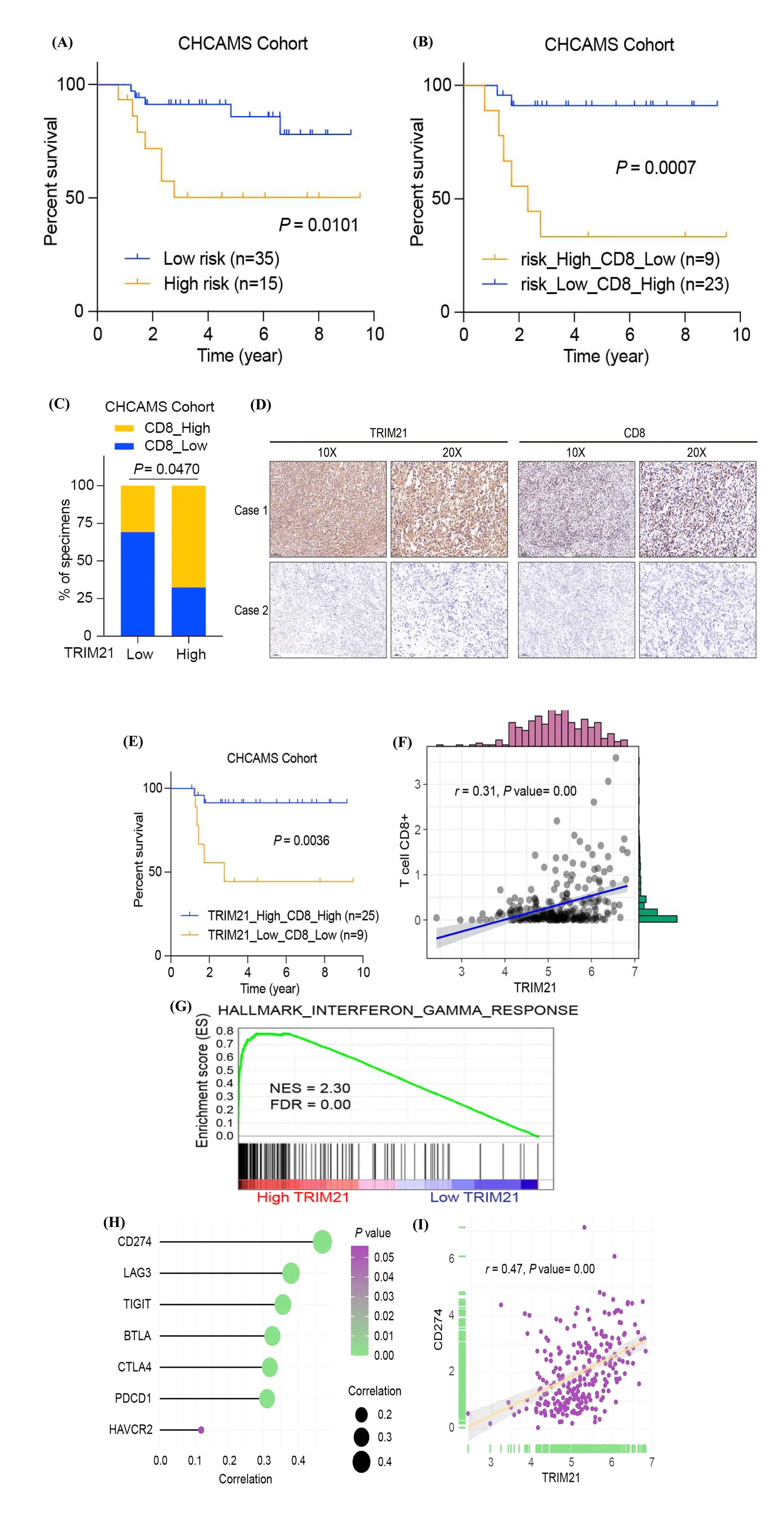
Validation of the model in an independent cohort and analyses for TRIM21. (**A**) Kaplan-Meier overall survival analyses of patients from different risk groups in CHCAMS cohort (n=50). (**B**) Kaplan-Meier overall survival analyses of patients stratified by both risk score and CD8 expressions. (**C**) The correlation between TRIM21 and CD8. (**D**) Representative immunohistochemistry images of TRIM21 and CD8 from a long-term survivor (Case 1) and a patient who deceased (Case 2). (**E**) Kaplan-Meier overall survival analyses of patients stratified by both TRIM21 and CD8 expressions. (**F**) The correlations between *TRIM21* and CD8^+^ T cells in TCGA-SARC patients. (**G**) Gene set enrichment analyses of the Hallmark interferon-gamma response in TCGA-SARC patients with different *TRIM21* expressions. (**H**) Correlations between *TRIM21* mRNA and prominent checkpoint genes. (**I**) The correlations between *TRIM21* and *CD274* (PD-L1) mRNA expressions. CHCAMS, Cancer Hospital, Chinese Academy of Medical Sciences; TCGA-SARC, The Cancer Genome Atlas sarcoma cohort.

To further corroborate the aforementioned findings, we used retrospectively collected tissue samples from 50 sarcoma patients who received surgical treatment at Cancer Hospital, Chinese Academy of Medical Sciences, and conducted immunohistochemistry assays to assess the expression levels of relevant proteins, with the representative IHC images of the proteins shown in Fig. (**S5A**). The clinical details of these patients are summarized in Table **S2**. H-scores of the proteins were evaluated, and risk scores were calculated. Since CD8 T cells were significantly correlated with the genes and risk score (Fig. **5A**), we also analyzed the correlation between protein levels of CD8 and these genes in our cohort. CD8 was significantly related to survival but was not significantly correlated with LRRC41, RNF125, UBE3D, or the risk score (Figs. **S5B-C**) in our cohort. In contrast, the expressions of RNF125 and TRIM21 (Fig. **S5C**) were significantly correlated with survival; the patients in the low-risk group had better OS (Fig. **6A**), and the model showed good performance in this cohort (Fig. **S5D**). Moreover, the combination of CD8 and risk score could better distinguish patients with different survival outcomes (Fig. **6B**).

One of the genes, TRIM21, is an E3 ubiquitin ligase that is well known to be involved in innate immunity and cancer proliferation [21-23]. Therefore, we focused on TRIM21. The expression of TRIM21 was positively correlated with that of CD8 in our cohort (Fig. **6C**), and representative images of two patients with different clinical outcomes were shown (Fig. **6D**). We have also observed that a higher expression of TRIM21 was significantly related to better patient survival (Fig. **S5C**). The combination of TRIM21 and CD8 could better distinguish patients’ OS (Fig. **6E**). Also, TRIM21 was correlated with CD8^+^ T cells in TCGA-SARC (Fig. **6F**). Enrichment analysis revealed that the Hallmark interferon-gamma response was enriched in patients with high TRIM21 expression (Fig. **6G**). Correlation analysis revealed that TRIM21 was positively correlated with several prominent checkpoint genes, including CD274, LAG3, TIGIT, BTLA, CTLA4, PDCD1, and HAVCR2 (Figs. **6H**-**I**).

### 3.6 Overexpression of TRIM21 Inhibits the Activity and Proliferation of Sarcoma Cells

**Fig. (7):**
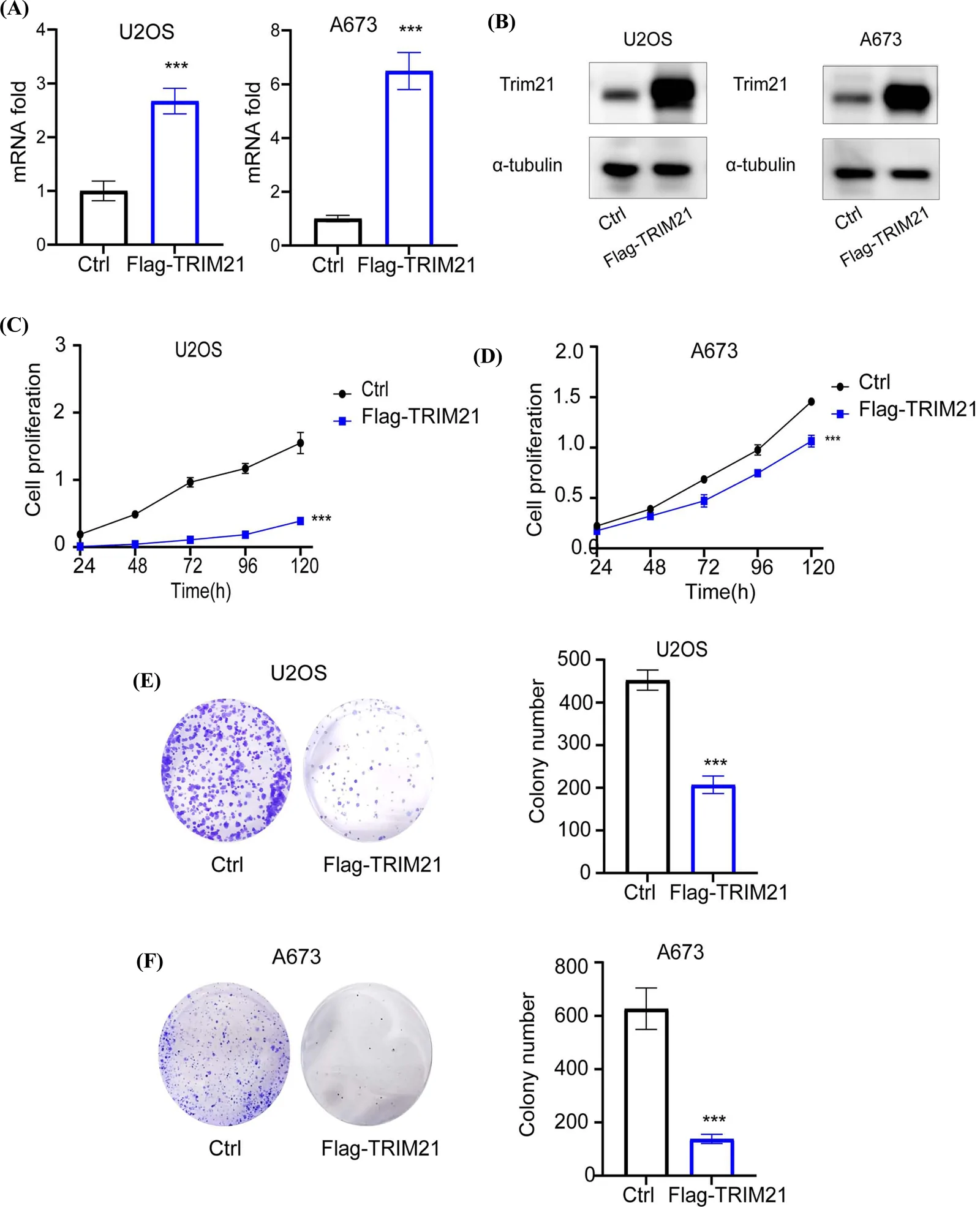
Overexpression of TRIM21 inhibits sarcoma cell proliferation *in vitro*. (**A, B**) Validation of TRIM21 overexpression in U2OS and A673 sarcoma cell lines by qRT-PCR (**A**) and western blotting (**B**). (**C, D**) CCK8 assays showing the viability of U2OS (**C**) and A673 (**D**) cells. (**E, F**) Colony formation assay of U2OS (**E**) and A673 (**F**) cells. ***p < 0.001.

To better understand the role of TRIM21 in sarcomas, we overexpressed TRIM21 using Flag-TRIM21 plasmids in the sarcoma cell lines U2OS and A673. The overexpression of TRIM21 in the cells was verified through qPCR and western blot analyses (Figs. **7A**, **B**). The experimental findings from the CCK8 experiment provided evidence that the overexpression of TRIM21 resulted in a decrease in cell viability, as seen in Figs. (**7C**, **D**). In addition, the results from colony formation assays demonstrated that the survival and proliferation capacities of sarcoma cells were significantly impaired when TRIM21 expression was enhanced, as seen in Figs. (**7E**, **F**). The results suggested that TRIM21 suppressed sarcoma progression and held promise as a potential therapeutic candidate for sarcomas.

## 4 DISCUSSION

Sarcomas are a group of invasive and heterogeneous mesenchymal tumors that are challenged by their rarity, intrinsic complexity, diagnostic technological complexity, and the limited impact of pathologists’ educational efforts [24]. Sarcomas have a poor response to current systemic therapies, which include only limited regimens, and are characterized by a considerably high rate of relapse or metastasis despite receiving optimal local treatment [2]. Specifically, sarcomas respond poorly to immunotherapy, and treatment responses are highly variable [2]. Despite their rarity, these tumors cause a significant loss of potential life years because they represent one of the most aggressive cancers in children and adolescents.

Therefore, numerous researchers have explored molecular resolutions to identify the intrinsic biological pathways and potential biomarkers for immunotherapy within each of the sarcoma subgroups. For example, Pankova *et al.* provided a comprehensive proteomic map of extracellular matrix and integrin adhesion networks in a broad range of sarcoma subtypes and developed a proteoglycan signature to predict patient prognosis [25]. Italiano A *et al.* performed an exploratory analysis of a multi-cohort phase 2 study, PEMBROSARC, and revealed that the abundance of intratumoral plasma cells was significantly associated with improved therapeutic outcomes in sarcoma patients receiving immunotherapy, highlighting the potential role of tertiary lymph structure in advanced soft tissue sarcoma as a predictive biomarker for immunotherapy [26].

In this study, URGs were integrated to identify different molecular clusters in sarcoma patients, and a prognostic signature was identified to stratify the risk levels of patients with sarcomas. Based on the prognostic values of the 138 URGs, patients from TCGA-SARC were classified into two clusters. The survival outcomes, enriched pathways, and tumor microenvironment characteristics were distinct between them. It is well-established that T cells infiltrate solid tumors and are associated with favorable clinical outcomes; therefore, immunotherapies have been developed to strengthen anti-tumor immunity based on this understanding [27, 28]. On the other hand, B cells are an important component of tertiary lymphoid structures, transient ectopic lymphoid aggregates where adaptive anti tumor responses occur, and are identified as a strong prognostic factor for survival in sarcomas [28, 29]. In an integrated analysis of the tumor microenvironment compositions of over 600 patients from four independent sarcoma datasets, subset C, characterized by the highest expression of genes specific to immune cell populations, was associated with favorable patient survival among all subtypes and could predict patients’ response to PD-1 blockade [29]. Similarly, in our analysis, patients in cluster 2 are characterized by better survival, and the enriched pathways identified by GSVA include interferon alpha response, interferon beta response, and complement, indicating an activated immune response. Further exploration of the tumor microenvironment revealed higher levels of B cells, CD8^+^ T cells, and a higher M1/M2 macrophage proportion, as well as a higher leukocyte and stromal fraction in cluster 2. Additionally, higher levels of MHC class II genes and immune checkpoints were observed in this cluster. Therefore, the integrated effects of higher immune infiltration, high expression levels of immune parameters, and a lower EMT score may contribute to the favorable survival outcomes observed in cluster 2. The effects, together with higher levels of checkpoints, indicated an immune-inflamed tumor microenvironment for this cluster, and implicated a higher susceptibility to immunotherapy.

To accurately evaluate the risk of individual patients based on ubiquitination, a four-gene prognostic signature was identified using URGs that were significantly correlated with patient survival in TCGA-SARC. The model could effectively distinguish between high- and low-risk patients from TCGA-SARC, TARGET-OS, and GSE17674. Likewise, the enriched pathways, immune infiltration, and EMT scores were distinct between the two risk groups. Patients in the low-risk group were correlated with higher expressions of most MHC class II genes, higher levels of CD8^+^ T cells, a higher M1/M2 macrophage ratio, and higher levels of checkpoints. The characteristics of low-risk group patients were akin to those in cluster 2 in our analyses and the ‘sarcoma immune Classes E’ group in the previous study [29]. These patients all experienced a favorable survival outcome. Therefore, the above results suggest that ubiquitination-related genes play a critical role in regulating the progression of sarcoma and are correlated with patients’ response to immunotherapy.

Among the four genes, RNF125 and TRIM21 were protective, while LRRC41 and UBE3D were risk factors for sarcoma patients. Among them, TRIM21 is an E3 ligase. It could bind to and target GRP78, which contributes to tumor growth and metastasis in osteosarcoma, facilitating ubiquitination and degradation [30]. Additionally, it was reported to balance the level of SNAI2 ubiquitination, working in conjunction with the deubiquitinase USP7, and thereby determining rhabdomyosarcoma vasculogenic mimicry, tumor cell proliferation, and migration [31]. Additionally, TRIM21 promotes the translocation of ANXA2 to the plasma membrane and induces autophagy in osteosarcoma cells [32]. In our study, TRIM21 was found to be correlated with a favorable prognosis in patients, and its overexpression suppressed the progression of sarcoma cells, identifying TRIM21 as a potential therapeutic target for sarcoma patients. For the other three genes, no relevant studies were reported in sarcomas. However, some have been reported in other tumors. For example, another E3 ligase, RNF125, has been identified as a tumor suppressor in hepatocellular carcinoma and inhibits its progression by targeting the SRSF1/ERK pathway [33]. Moreover, RNF125 can interact with PD-L1 and induce K48-linked ubiquitination of it for degradation [34]. LRRC4 is a membrane-bound transcription factor that can interact with the enhancer element of the RAP1GAP gene to promote its transcription and suppress glioblastoma cell migration by modulating cell contraction and polarization [35]. It could act as an autophagy inhibitor and restore the sensitivity of glioblastomas to temozolomide. Additionally, LRRC4 could inhibit the proliferation of glioblastoma cells by promoting the generation of circCD44 [36, 37]. As a HECT-like E3 ligase, UBE3D stabilizes CPFS73 protein by preventing it from being ubiquitinated and degraded by the proteasome, thus promoting breast cancer stemness [38]. In prostate cancer, UBE3D binds to and ubiquitinates the steroidogenic enzyme 3βHSD1, targeting it for proteasomal degradation. This suppression of intracrine dehydroepiandrosterone metabolism negatively regulates tumor metabolic heterogeneity, positioning UBE3D as a key determinant for patient stratification and targeted therapy [39]. The combination of these genes may arise from innate biological processes, suggesting novel therapeutic strategies for sarcoma patients. The specific molecular mechanisms driving this combination, however, need to be elucidated in future research.

The EMT is defined as the reprogramming of cells from an epithelial phenotype into a mesenchymal phenotype and is characterized by the loss of intercellular interactions and a lack of polarity, as well as an increase in extracellular matrix interfaces, facilitating invasion and migration [40]. In this study, we observed that the Hallmark EMT pathway was enriched in cluster 1 and the high-risk group. Consistently, a higher EMT score was identified in them. Since sarcomas are malignant tumors arising from mesenchyme, they are already equipped with mesenchymal phenotypic features and do not need to undergo the EMT process [41]. A possible explanation is that the metastable phenotype between the epithelial and mesenchymal stages enables sarcomas to undergo the EMT process under specific conditions, contributing to their aggressive clinical behavior [41]. These behaviors then lead to a worse clinical outcome.

There are several limitations. First, given the retrospective nature of the study, it primarily utilized publicly available datasets. Second, similar to other studies [1, 25, 29, 42, 43], the inherent heterogeneity of sarcomas and their microenvironment were not fully addressed, and the prognostic values of the model have just been verified in a small cohort due to the rarity of samples, which may reduce the generalizability of our results to all sarcoma patient subsets. The prediction for response to immunotherapy was also not validated, due to the lack of immunotherapy cohorts. For further validation, a large-sample-size prospective study is needed. Lastly, some of the genes in the signature have rarely been reported and are worthy of further experimental research.

## CONCLUSION

In conclusion, the study successfully identified distinct ubiquitin-related clusters in sarcoma patients, which were closely associated with patient survival outcomes and tumor microenvironment. Based on the clusters, we identified an effective prognostic gene signature and observed that a high risk score is associated with worse OS and impaired immunotherapy efficacy. The prognostic value of the signature was validated at the protein level using an independent cohort from our center, and the suppressive role of TRIM21 was confirmed in cellular experiments. In a tumor with a considerably high relapse rate and a low response rate to immunotherapy, these results take on particular importance. The signature could be useful for guiding individual patient stratification and may help identify suitable patients for future clinical trials, providing clues for future research.

## Data Availability

The datasets used in the current study are available from the corresponding author on reasonable request.

## LIST OF ABBREVIATIONS

ANXA2: Annexin A2
BTLA: B and T lymphocyte attenuator
CCK8: Cell Counting Kit-8
CD8: Cluster of Differentiation 8
CD274: Programmed Death-ligand 1
CHCAMS: Cancer Hospital Chinese Academy of Medical Sciences
CIBERSORT: Cell-type Identification By Estimating Relative Subsets Of RNA Transcripts
CPFS73: Cleavage and Polyadenylation Specificity Factor 73
CTLA4: Cytotoxic T-lymphocyte-associated Protein 4
DMEM: Dulbecco's Modified Eagle Medium
EMT: Epithelial-Mesenchymal Transition
EPHX1: Epoxide Hydrolase 1
ERK: Extracellular signal-Regulated Kinase
FBW7: F-box/WD Repeat-containing Protein 7
GEO: Gene Expression Omnibus
GPX4: Glutathione Peroxidase 4
GRP78: Glucose-regulated Protein 78
GSEA: Gene Set Enrichment Analysis
GSVA: Gene Set Variation Analysis
HAVCR2: Hepatitis A Virus Cellular Receptor 2
IHC: Immunohistochemistry
Indel: Insertion/Deletion
LAG3: Lymphocyte-activation Gene 3
LRRC41: Leucine Rich Repeat Containing 41
MHC: Major Histocompatibility Complex
MSigDB: Molecular Signatures Database
NMF: Non-negative Matrix Factorization
OS: Overall Survival
PD-1: Programmed Cell Death Protein 1
PDCD1: Programmed Cell Death 1
PRMT5: Protein Arginine Methyltransferase 5
qRT-PCR: Quantitative Real-Time Polymerase Chain Reaction
RNF125: Ring finger protein 125
ROC: Receiver Operating Characteristic
SARC: Sarcoma
SNV: Single Nucleotide Variant
SRSF1: Serine/arginine-Rich Splicing Factor 1
TARGET: Therapeutically Applicable Research to Generate Effective Treatments
TCGA: The Cancer Genome Atlas
TCR: T-cell Receptor
TGF-β: Transforming Growth Factor Beta
TIGIT: T cell Immunoreceptor with Ig and ITIM Domains
TIMER: Tumor Immune Estimation Resource
TRIM21: Tripartite Motif-containing Protein 21
TXNIP: Thioredoxin-interacting Protein
URGs: Ubiquitin-related Genes
USP7: Ubiquitin-specific Protease 7

## STANDARDS OF REPORTING

STROBE guidelines were followed.
