# Supplementary material for: Ubiquitination-based Classification and a Prognostic Signature Identify the Role of TRIM21 in Sarcoma Progression
Source: Curr Med Chem. 2026 Jan 15;33(16):3246–71. doi: 10.2174/0109298673388379251113061745 (PMC13555846; doi:10.2174/0109298673388379251113061745)
Supplement: Supplementary file 1 [file CMC-33-16-3246_SD1.zip › CMC-33-16-3246_SD1/CMC-33-16-3246_SD1.pdf]

Supplementary Material

Ubiquitination-based Classification and a Prognostic Signature Identify the Role of TRIM21 in Sarcoma Progression

Lin Zhang[1,#], Weihao Lin[2,3,#], Jinhui Liu[3,#], Yuheng Hong[3], Zheng Cao[4], Zhentao Yu[1,*], Xiaoli Feng[4,*] and Yibo Gao[5,6,7,8,*]

[1]Department of Thoracic Surgery, National Cancer Center/National Clinical Research Center for Cancer/Cancer Hospital and Shenzhen Hospital, Chinese Academy of Medical Sciences and Peking Union Medical College, Shenzhen, 518116, China; [2]Department of Thoracic Surgery, Nanfang Hospital, Southern Medical University, Guangzhou, 510515, China; [3]Department of Thoracic Surgery, National Cancer Center/National Clinical Research Center for Cancer/Cancer Hospital, Chinese Academy of Medical Sciences and Peking Union Medical College, Beijing, 100021, China; [4]Department of Pathology, National Cancer Center/National Clinical Research Center for Cancer/Cancer Hospital, Chinese Academy of Medical Sciences and Peking Union Medical College, Beijing, 100021, China; [5]Central Laboratory & Shenzhen Key Laboratory of Epigenetics and Precision Medicine for Cancers, National Cancer Center/National Clinical Research Center for Cancer/Cancer Hospital & Shenzhen Hospital, Chinese Academy of Medical Sciences and Peking Union Medical College, Shenzhen, 518116, China; [6]Laboratory of Thoracic Oncology & Department of Thoracic Surgery, National Cancer Center/National Clinical Research Center for Cancer/Cancer Hospital, Chinese Academy of Medical Sciences and Peking Union Medical College, Beijing, 100021, China; [7]State Key Laboratory of Molecular Oncology, National Cancer Center, National Clinical Research Center for Cancer, Cancer Hospital, Chinese Academy of Medical Sciences and Peking Union Medical College, Beijing, 100021, China; [8]Laboratory of Translational Medicine, National Cancer Center/National Clinical Research Center for Cancer/Cancer Hospital, Chinese Academy of Medical Sciences and Peking Union Medical College, Beijing, 100021, China

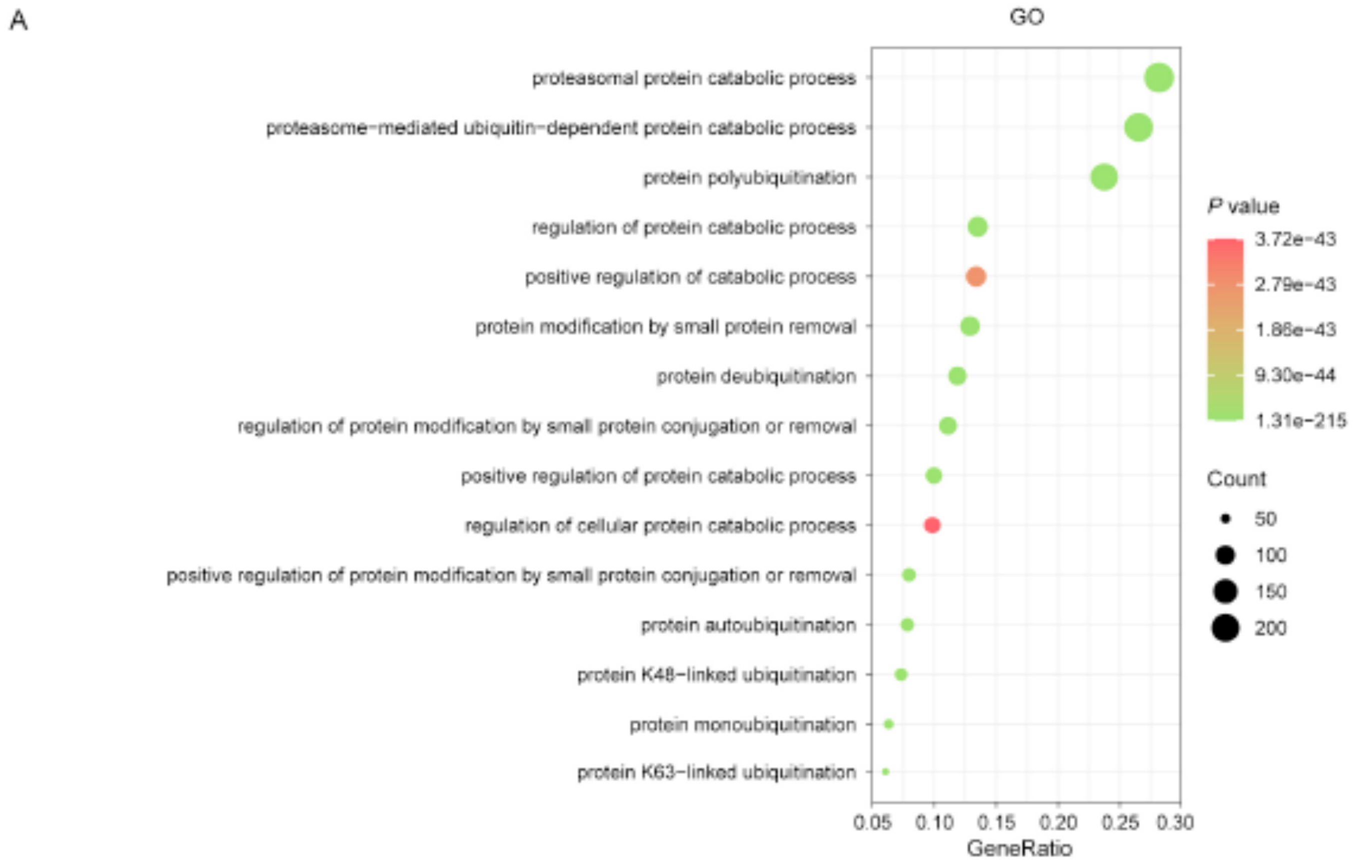

Fig. (S1). Contd….

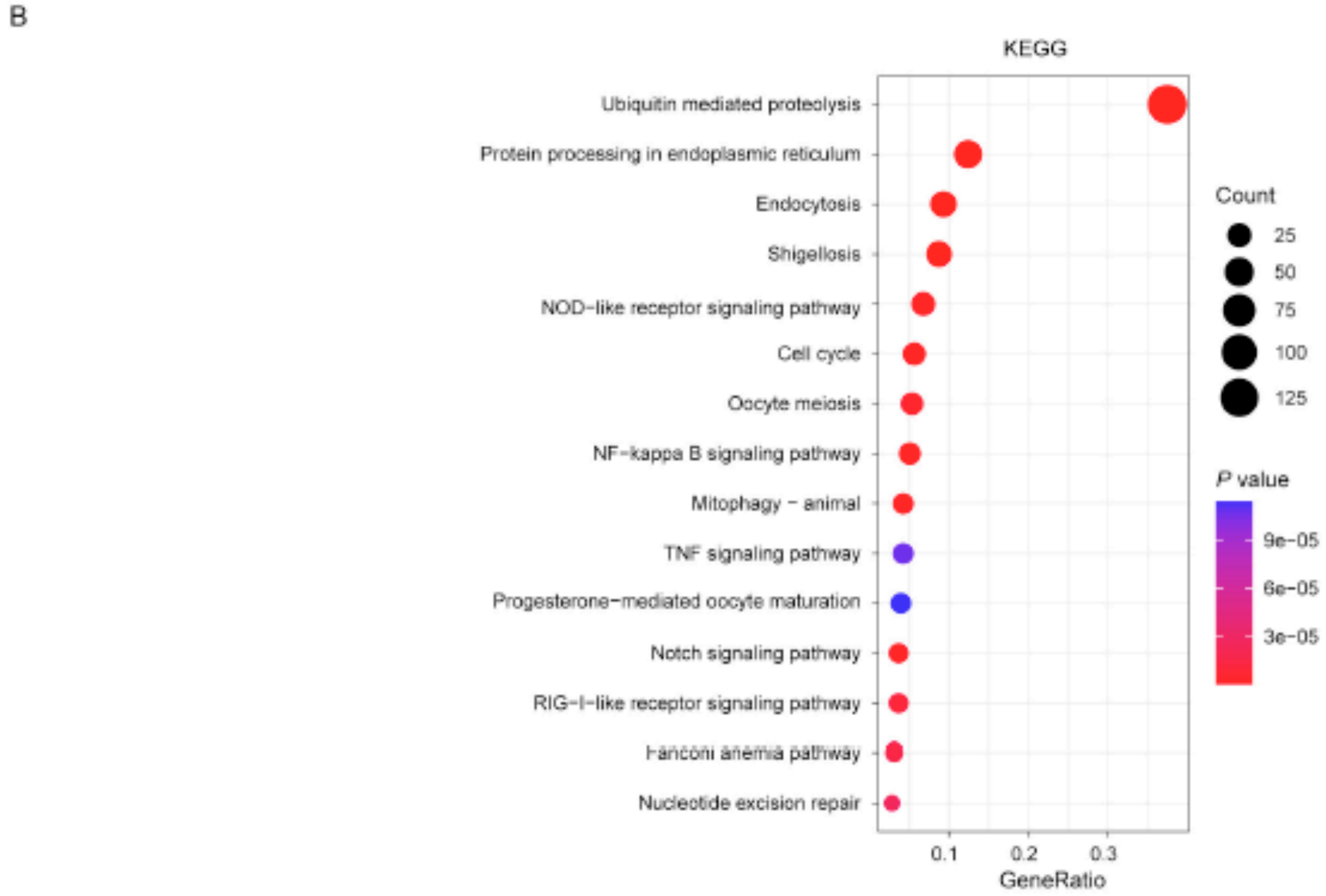

Fig. (S1). Enrichment analysis of the ubiquitin-related genes. **A** Analysis of Gene Ontology terms. **B** Analysis of Kyoto Encyclopedia of Genes and Genomes pathways.

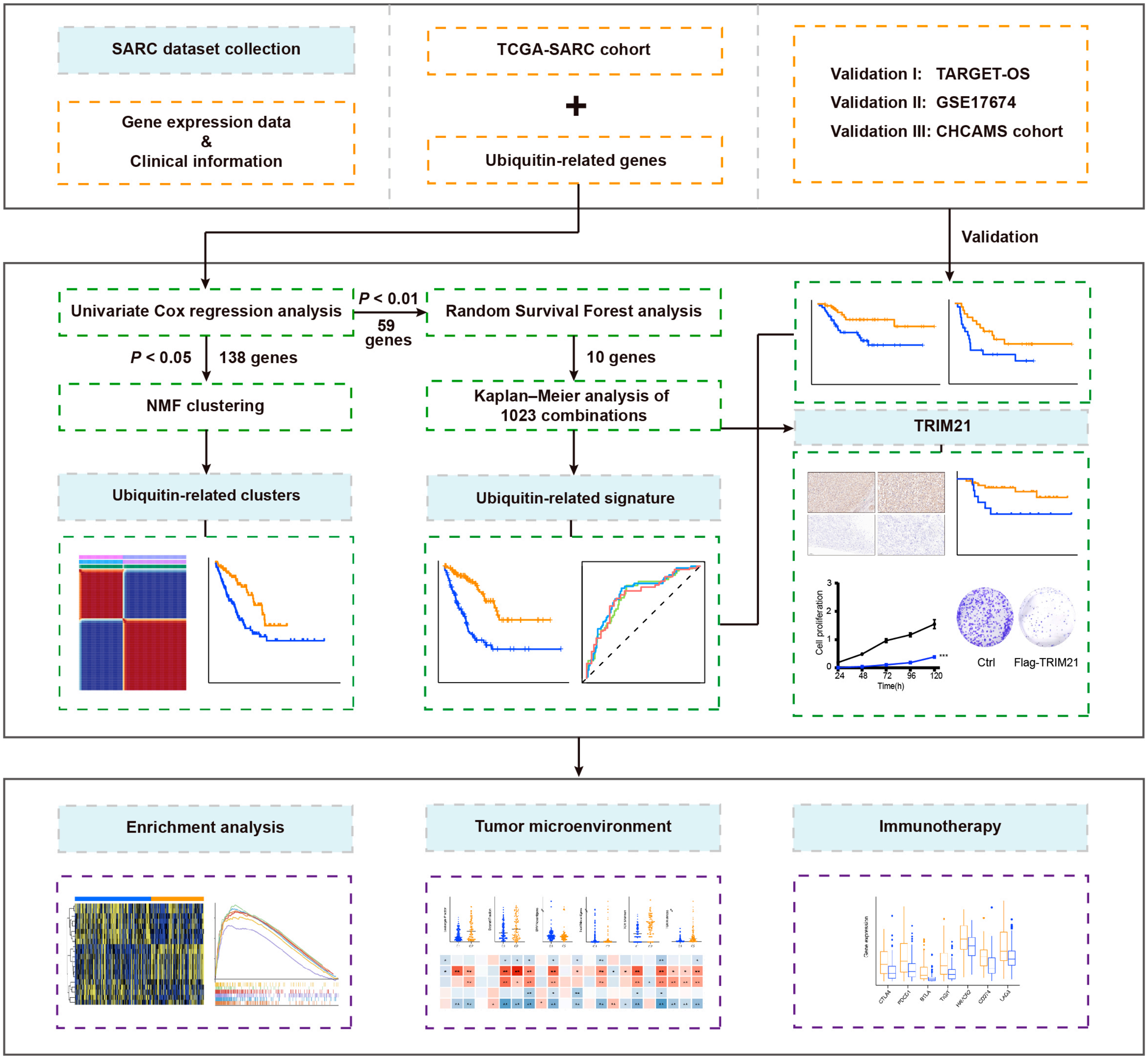

Fig. (S2). Flow chart of this study. SARC, sarcoma; TCGA-SARC, The Cancer Genome Atlas sarcoma cohort; TARGET-OS, the osteosarcoma cohort from the TARGET database; CHCAMS, Cancer Hospital, Chinese Academy of Medical Sciences; NMF, nonnegative matrix factorization.

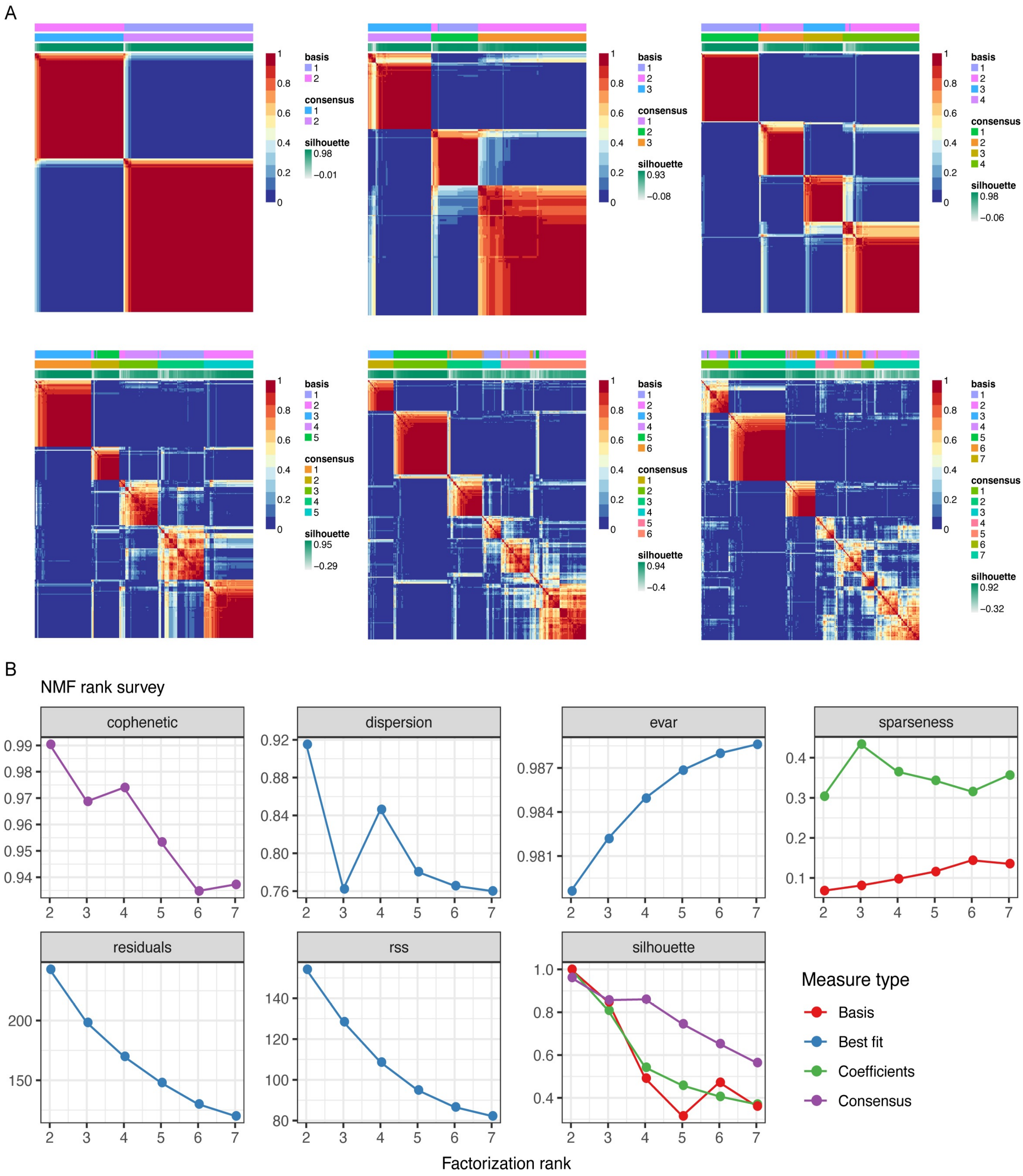

Fig. (S3). Identification of ubiquitin-related clusters. **A** Clustering matrix of TCGA-SARC samples for k = 2 to k = 7. **B** The relationships between cophenetic, dispersion, evar, sparseness, residuals, rss, silhouette coefficients and the number of clusters. TCGA-SARC, The Cancer Genome Atlas sarcoma cohort.

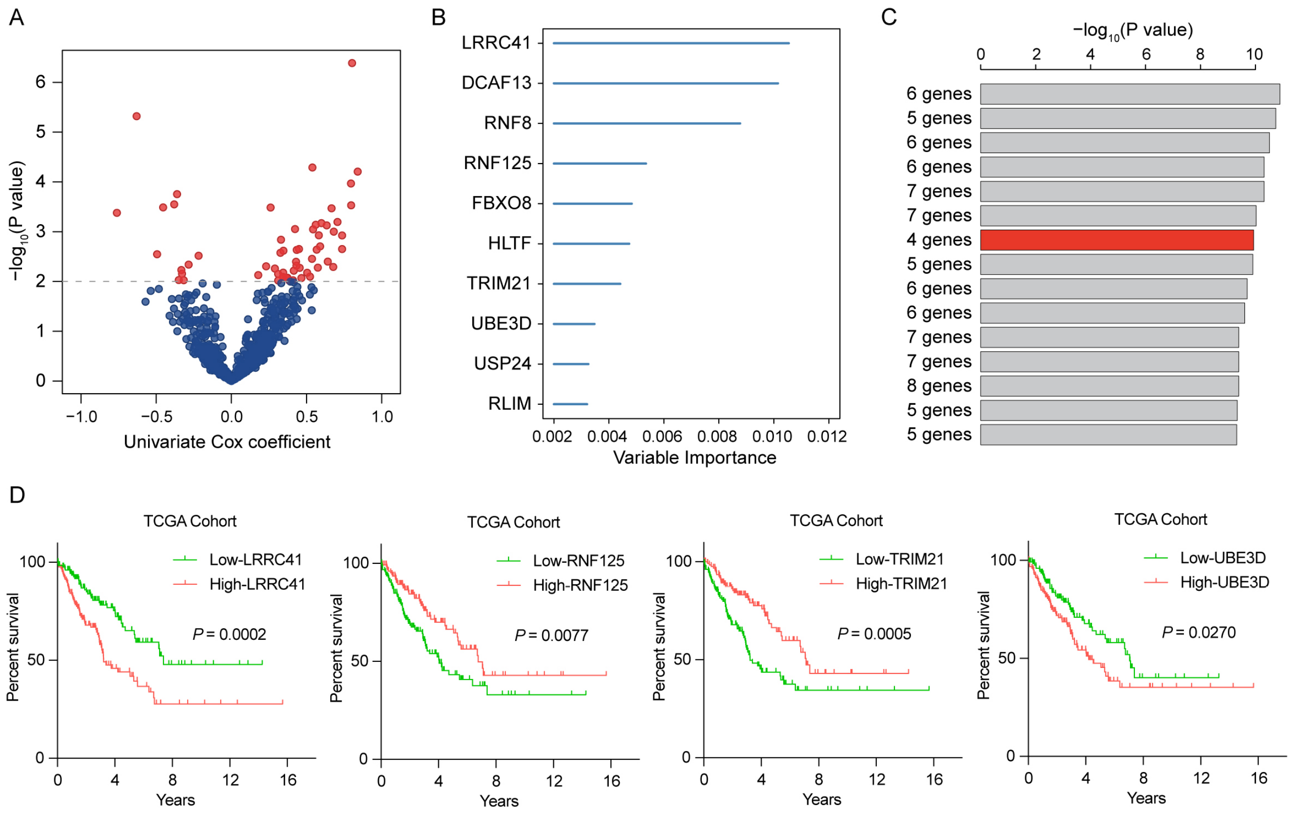

Fig. (S4). Construction of the ubiquitination-related prognostic signature. **A** Volcano plot showing univariate analyses of the ubiquitin-related genes in TCGA-SARC. **B** 10 genes ranked by importance that are screened by random survival forest analysis. **C** The Log-rank *p* values of the top 10 combinations for predicting overall survival. **D** Kaplan-Meier overall survival analyses of TCGA-SARC patients stratified by the expression of each individual signature gene. TCGA-SARC, The Cancer Genome Atlas sarcoma cohort.

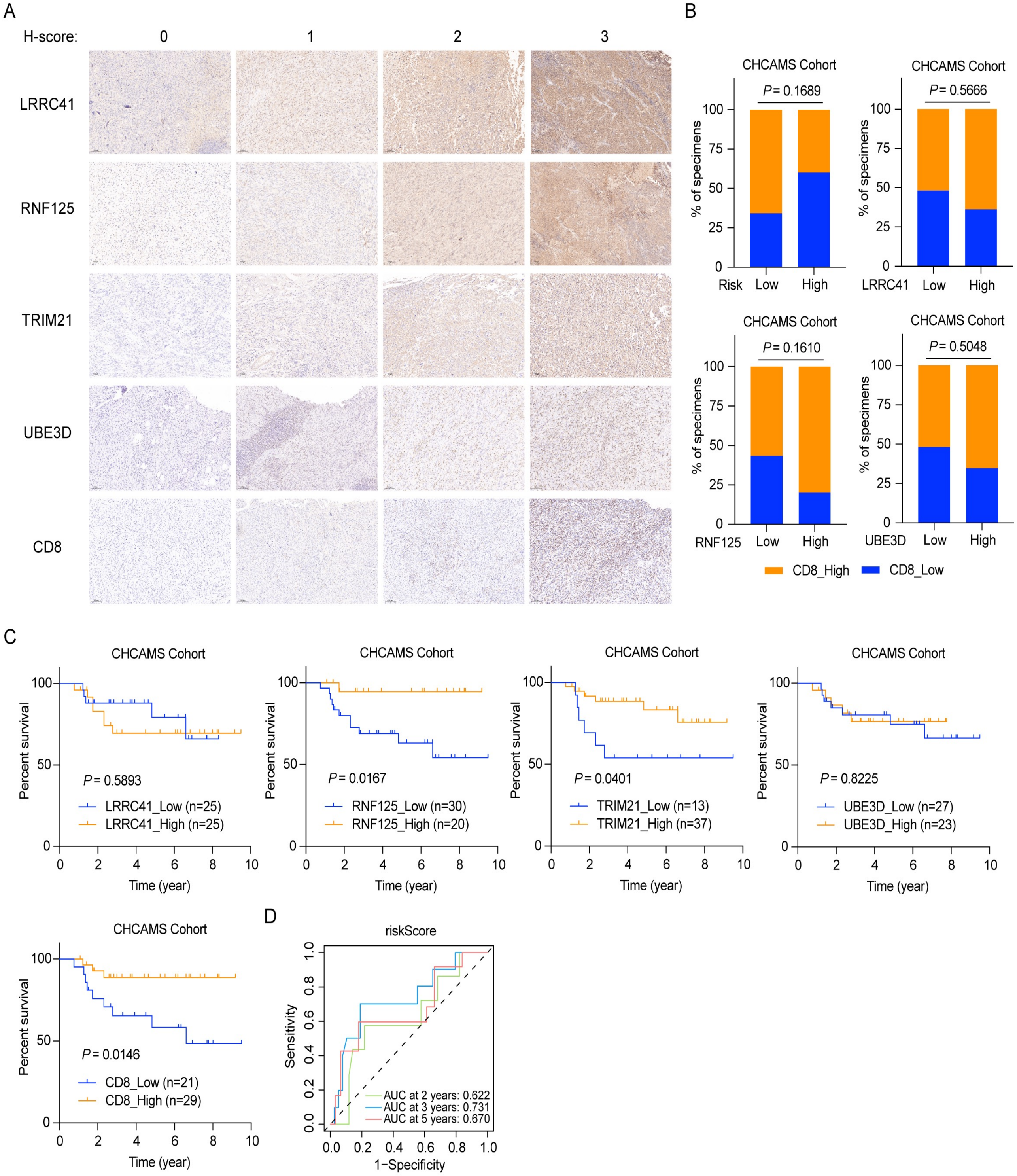

Fig. (S5). Validation of the model in the CHCAMS cohort. **A** Representative immunohistochemistry images of LRRC41, RNF125, TRIM21, UBE3D, and CD8. **B** Correlations between dichontomized CD8 and risk, LRRC41, RNF125, and UBE3D. **C** Kaplan-Meier survival analyses for LRRC41, RNF125, TRIM21, UBE3D, and CD8 in CHCAMS cohort. **D** Receiver operator characteristic curve of risk score for predicting overall survival in the CHCAMS cohort. CHCAMS, Cancer Hospital, Chinese Academy of Medical Sciences.
